# The Role of Family on Hookah Smoking Initiation in Women: A Qualitative Study

**DOI:** 10.5539/gjhs.v7n5p1

**Published:** 2015-02-24

**Authors:** Azam Baheiraei, Shirin Shahbazi Sighaldeh, Abbas Ebadi, Roya Kelishadi, Reza Majdzadeh

**Affiliations:** 1 Community-Based Participatory Research Center, Iranian Institute for Reduction of High-Risk Behaviors, Tehran University of Medical Sciences, Tehran, Iran; 2 Department of Reproductive Health, School of Nursing and Midwifery, Tehran University of Medical Sciences, Tehran, Iran; 3 Behavioral Sciences Research Center (BSRC) & Nursing Faculty, Baqiyatallah University of Medical Sciences, Tehran, Iran; 4 Child Growth and Development Research Center, Isfahan University of Medical Sciences, Isfahan, Iran; 5 School of Public Health and Knowledge Utilization Research Center, Tehran University of Medical Sciences, Tehran, Iran

**Keywords:** hookah, narghile, qualitative research, smoking, shisha, tobacco, water pipe, women

## Abstract

Hookah smoking has recently emerged as a popular alternative to cigarette smoking particularly among young adults and women. This study focused on the role of family members’ smoking behaviours as a possible risk factor for initiation of hookah smoking in women. 36 in-depth interviews were conducted with Iranian women of diverse ages for understanding the factors contributing to the initiation of hookah smoking. Four main themes were identified from the data. This study focused on the role of family as a facilitator for hookah smoking initiation. The results of this study indicate that the entry of hookah into homes can be effective in the spread of hookah smoking among adult and young women, in three ways: Girls’ participation in the preparation of hookah and the frequent observation of people who smoke hookah at home can be effective in hookah smoking initiation among young girls; the husband of a young woman has an important role in the initiation of smoking hookah; when parents invite children to smoke hookah at home, in order to protect them against public censure, the mother (a middle aged woman) may intend to start smoking hookah. Therefore, tobacco use prevention interventions should be focused on targeting the family as well.

## 1. Introduction

Cigarette smoking remains the most prevalent form of tobacco use in girls and in women of reproductive age globally but use of non-cigarette forms of tobacco, most notably hookah smoking, is prevalent or gaining in popularity in many parts of the world (England et al., 2010; Warren et al., 2009). Women have been extensively targeted in tobacco marketing dominated by themes of an association between social desirability, independence, weight control and smoking messages conveyed through advertisements featuring slim, attractive, and athletic models (American Lung Association, 2014). Hookah factors that promote its popularity may include its social acceptance as part of cultural heritage, easy availability, attractive designs, and the availability of flavored aromatic tobacco called “Massal”. In some Arab countries, hookah smoking is considered less of a social stigma than cigarette smoking, and is also associated with less gender differentiation (Amin, Amr, Zaza, & Kaliyadan, 2012).

The practice of hookah smoking, also known as: waterpipe, narghile, hubble bubble, goza and shisha, originated in India during the early 1600’s (Asfar, Ward, Eissenberg, & Maziak, 2005; Eissenberg & Shihadeh, 2009), but its use was declining until recent years when it witnessed a boom in popularity and this smoking habit reached a quarter of some groups in the Middle Eastern region (EMR), affecting youth and women among others (Asfar et al., 2005). Even in some regions hookah consumption among women is higher than men’s intake. In a Lebanese study, for example, the majority of hookah smokers were women (Riachy et al., 2008). Spreading from the Eastern Mediterranean region, hookah use is now common in Western countries including the UK and the USA (Maziak, 2011).

Research on the health effects of hookah use is in its infancy relative to cigarettes, but studies conducted so far indicate both short-term and longer term health consequences similar to those of cigarettes (Afifi, Yeretzian, Rouhana, Nehlawi, & Mack, 2009). Boskabady, Farhang, Mahmodinia, Boskabady and Heydari (2012) reported that hookah smoking can negatively affect lung function similar to the effects of deep inspiration cigarette smoking and Raad et al. (2011) introduced hookah smoking as a potential cause of Chronic Obstructive Pulmonary Disease (COPD). According to a systematic review hookah smoking is significantly associated with lung cancer, respiratory illness, periodontal disease and low birth-weight (AkI et al., 2010). In spite of these deleterious health effects, hookah smoking is widely believed to be a less harmful form of tobacco smoking (Maziak, Eissenberg, & Ward, 2005a) and a safer alternative to cigarette smoking (Primack et al., 2008).

### 1.1 Background of Hookah Smoking in Iran

The results of the last national Survey of Risk Factors of Non-Communicable Diseases (SuRFNCD-2007) in Iran showed that more than half of tobacco smoking women smoke tobacco using hookah (Pasha Meysami, Ghalet, Haghazali, & Asgari, 2010). Similar to the Arab countries, in the Iranian community, there is less of a stigma associated with hookah (Kelishadi et al., 2007) and hookah smoking is more acceptable than cigarette smoking, especially among girls (Sarafzadegan et al., 2010). Thus, hookah smoking in Iranian women has increased over the rate of cigarette consumption (Allaeikhorram, Kadivar, Mohammadkhani, Sarami, & Allaeikhorram, 2011) and increased prevalence of hookah smoking in Iranian women has lead to reduced differences in smoking patterns among women and men (Taraghijah, Hamdiye, & Yaghoubi, 2010).

In Iran, usually the elderly, most notably women used hookah, however with a revival in recent years hookah smoking is also being casually practiced by youth for fun in traditional teahouses (Kelishadi et al., 2007). Because hookah smoking is seen in women in all ages, investigating the factors influencing the use of hookah smoking should consider in all age groups.

So far, most Iranian quantitative studies associated with smoking have focused on cigarette smoking (Rahmanian, Jafarzadeh, & Khalouei, 2011; Mojahed & Bakhshani, 2005) and only a few studies, in recent years, have addressed the causes of hookah smoking in both genders (Roohafza, Sadeghi, Shahnam, Bahonar, & Sarafzadegan, 2011; Sahaby, Divsalar, & Nakhaee, 2011). But no study has been done on the factors associated with hookah initiation in women separately from men, despite the fact that risk factors for smoking may vary by gender (WHO, 2010) and also the factors related to hookah initiation and maintenance may be different in both genders (Roohafza et al., 2011) despite the fact that such information is essential for developing health promotion initiatives and interventions that specifically address women and girls.

To date, much research has focused on the role played by families in cigarette smoking behaviour. In these studies, the influence of family has been reported as an important factor in the initiation and continuation of smoking (Mousavi, 2004; Oh et al., 2010; Moeini & Verdipour, 2011; Kasiri, Raafiei, Haghighizadeh, & Kazemzadeh, 2012), but there have been no studies conducted on how family influences the initiation of hookah smoking in women.

### 1.2 Aim

This study is part of a larger exploratory mixed methods study which aims to explore the influence of different factors on the initiation of hookah smoking in women. Because of the large volume of qualitative data obtained, in this article only part of the qualitative phase about the role of family members and husbands’ hookah smoking behaviour is discussed as a possible risk factor for initiation of hookah smoking among women.

## 2. Methods

### 2.1 Participants and Data Collection

In this descriptive qualitative study, 36 in-depth interviews were conducted with Iranian women of diverse ages for understanding the factors contributing to the initiation of hookah smoking. Qualitative methods are the preferred method for exploring people’s perceptions of the factors that influence health behaviors and understanding the context in which choices are made. In this research, a qualitative approach was adopted using conventional content analysis of semi-structured interviews in Tehran in 2012. In conventional content analysis coding categories are derived directly from the text data, without the guidance of a theory for initial codes, as in directed approach (Hsieh & Shannon, 2005). Participants included women who were current, former or ever user of hookah and hookah smoking status was established in accordance with the criteria set by Maziak, Ward, Afifi Soweid, and Eissenberg (2005b) for hookah smoking. Current hookah use was defined as having used hookah at least once in the previous month and former hookah use was defined as having used hookah at least once a month for three consecutive months in the past and have left it now. Ever hookah use was defined as having ever actively inhaled smoke from hookah even one or two inhalations.

Women of different age groups were able to participate in this study. Unlike in many Western countries where hookah smoking has gained popularity in recent decades, in Iran hookah has been used over the centuries. In the past, hookah was usually smoked by certain adult men and women but now all adults and youth smoke hookah. For this reason the age limit for participant’s entry into the study was not considered.

Participants were sampled purposively from universities, hospitals, through home visits, leisure centers and cafes following a snowball technique where one person would put the researcher in touch with her friends, colleagues, and other contacts who smoked hookah.

Semi-structured interviews were conducted by a researcher who experienced in qualitative method of research. However, there is less of a stigma associated with hookah than with cigarette smoking in the Iranian women but women are simply not yet ready to talk about their hookah and preferred not to discuss it in any public situation. As experienced in this study, some women who were introduced to the researcher refused to participate in the interview because of the opposition of their parents/husband or because of personal disagreement with sound recording or appointment. If they accepted to talk, they preferred to do it in a private space rather than in a formal gathering like focus group discussion. Thus, only individual interviews were used to collect data in this study.

The researcher explained the purposes of the research, that participation is voluntary and that they may stop the interview at any time and that confidentiality of records is ensured. Following their approval to participate in the interview, their consent, verbal or written, to record the interview was also obtained.

Participants were asked to describe their experience of the first use of hookah and what factors influence the initiation of smoking. A demographic and pattern of hookah use questionnaire which was developed by the researchers was used before each interview. The questionnaire included questions about age at time of interview, age of first use of hookah, occupation, location, ethnicity, marital status and current or past use of hookah. Interviews were based on topic guides, including a series of broad interview questions which the researcher considered to explore and probe with the interviewee. The questions that were used to guide the participants in the interviews include: “Why do people start to smoke hookah?” In what circumstances and where did you smoke hookah for the first time?” “Who was with you at the first session of hookah smoking?” and, “Did anyone encourage you to smoke hookah?”

Data collection was stopped when data saturation was reached, i.e. no new themes or ideas were being generated during the discussions.

### 2.2 Data Analysis

Interviews were conducted in Farsi and translated into English by an accredited institution. They were recorded and took from twenty minutes to one hour and a half. All interviews were audio-taped and transcribed verbatim with participants’ permission and then coded by researcher. Coding is an essential step in the organization, processing and analysis of qualitative information and paves the way for the interpretation phase. Development of codes and themes was inductive and arose from the interviews. We analyzed the transcripts by identifying emergent themes using constant comparison of the interview transcripts. Data generation and analysis continued until no new themes or ideas were emerging. The final coding scheme consisted of 4 themes and 20 sub-themes. Code management was done with the help of MAXQDA10 software which is one of the best qualitative data analysis tools in the world (http://www.maxqda.com/products/great-reasons-to-use-maxqda).

### 2.3 Methodological Considerations

Credibility and conformability was enhanced through member checking (in this case, the transcripts and codes extracted from the interviews were returned to several interviewees to verify their authenticity), and validation of emerging codes and categories in subsequent interviews, and also debriefing with two supervisors. To establish inter-transcripts reliability, two experts carried out a second review. Almost all of the transcripts, codes and categories were rechecked and there was strong agreement among the study team and advisors. Cases of disagreement were discussed to reach a final consensus and resolved by discussion among the team members or by going back to the original transcripts.

### 2.4 Ethical Consideration

Ethical approval was granted by the Ethics Committee of Tehran University of Medical Sciences.

## 3. Results

From 49 women who were invited, 36 women agreed to participate in our study. 31 participants were current users of hookah, 1 participant was ever user of hookah and 4 participants were former users of hookah. The age of participants ranged from 15 to 51 years old, with a median age of 24 yrs. Age at onset of smoking hookah ranged from 7 to 42 years, with a median age of 25 yrs. Participants were married, single or divorced women belonging to different geographic regions of Tehran and were from different ethnic sub-groups. Most participants had a diploma or academic degree. Almost half of the women were employed.

This manuscript will describe the family members’ role on the hookah smoking initiation in women.

### 3.1 Individual, Familial and Social Facilitators

#### 3.1.1 The Role of Family and Relatives

Girls whose mothers smoke hookah or whose relatives used to smoke in family parties and their family has a tradition of smoking hookah may start hookah smoking from home, usually in a family gathering. These girls may start smoking hookah from a very young age. In this study, 56% of participants said that they had started smoking hookah with their family members and some had experienced hookah for the first time during childhood. When engaged in hookah use as a family tradition, mothers sometimes wanted their daughters to prepare a hookah. When preparing the hookah for their mother, the girl may take at least one or two puffs. In this situation, the girl is impressed by the traditional values of family. One participant said:

*“I was about 15 years old. My mom, her sister and her sisters-in-law, all of them, used to get together and smoke hookah. My mother usually asked me to go to the kitchen and prepare the hookah for them. When I went through the kitchen to my mom, I took a few puffs…I wondered what hookah was…”* (Current smoker, 48 years old, first experience at age 16)

Based on the study results, parents who smoke hookah may directly or indirectly affect their daughters to start smoking hookah. However, parents usually prohibit their children from smoking the hookah in childhood and adolescence. They want to protect their children and believe that this prohibition is due to the young age of the child or fear of the consequences of smoking hookah outside the home, such as stimulation for drug abuse.

*“Parents prefer their children to experience (their first) hookah (trial) with them (At home, not with their friends, or in hookah cafes). This is because they are afraid their kids might try something plus hookah (such as drugs) with their friends (or when they are in the cafes). It doesn’t matter whether it’s a girl or a boy (Parents are worried for them).”* (Current smoker, 24 years old, first experience at age 22)

Nevertheless, some parents have a neutral stance against their children’s curiosity to smoke hookah. It does not matter to them whether the kids smoke hookah or not. Some parents disagree with their children temporarily, while others encourage them to smoke.

*“I was too young the first time I smoked hookah. My uncle and my dad were smoking hookah. I wondered what it was…Then my dad taught me how to blow into the hookah tube.”* (Current smoker, 24 years old, first experience at age 9)

Sometimes the first hookah use occurs among a gathering of friends, outside the home, but this interest is rooted in family culture. An 18-year-old girl had started smoking hookah with her friends, but her interest in hookah smoking was related to her father’s and brother’s smoking at home. In this regard, she said:

*“The first time I smoked hookah, I was in a traditional restaurant (Sofre-khaneh) with a friend. Because my father and my brother used to smoke hookah at home, I wanted to try it but they wouldn’t let me (to smoke). Now, I smoke hookah easily, even in their presence. When the hookah comes, I do not know what happens, but whatever it is, the whole atmosphere changes and becomes happier. Not only in family gatherings, everywhere…”* (Current smoker, 18 years old, first experience at age 13)

Although the experiences of hookah use in childhood may be a short-term experience and children just try it a few times, they may keep the memory of this experience in their mind and start smoking again in the future and continue it:

*“When I started smoking hookah, I was 6 or 7 years old, in Mashhad. All adults smoked hookah, I smoked too. In my family, we don’t interfere in others’ business. I felt bad when I smoked. I puked. After that I didn’t smoke hookah. I began smoking hookah again when I was at middle school. When I was a university student, I was totally addicted.”* (Current smoker, 32 years old, first experience at age 7)

In some Iranian families, hookah smoking is a way to welcome guests. As a result, even families who do not smoke may buy a hookah to welcome their relatives and thus, they will gradually start smoking hookah in the family like their guests. One participant said, quoting her daughter:

*“It’s bad to ask everyone who comes to our home to bring their hookah. Let’s buy one.”* (Current smoker, 48 years old, first experience at age 16)

At first, they may only smoke hookah with their guests, or in family gatherings, and then they can even use it alone. In these cases, typically all family members may be at risk to start smoking hookah and therefore, in this circumstance, girls’ tendency toward hookah use is not met with a negative response or opposite reaction.

Parents who only smoke cigarettes can also indirectly influence children to start smoking hookah:

*“My father can never tell me not to smoke hookah because he himself smokes cigarettes.”* (Current smoker, 24 years old, first experience at age 19)

In some families, parents do not smoke cigarettes or hookah but their children smoke hookah and cigarettes. In these cases, hookah smoking is often learned through peer relationships.

*“Our parents (the parents of my cousins) don’t smoke cigarettes but all kids smoke hookah. I can say almost all the kids smoke hookah, more girls than boys. The first time, I was with my cousins my uncle’s son brought his hookah, and we all smoked.”* (Current smoker, 28 years old, first experience at age 25)

As a mother can have an impact on hookah use by her children, children can also be effective in the hookah smoking of their mothers. Thus, some women begin to smoke hookah because of their children (daughter or son). Four of the participants that were over 40 years old stated their reasons for starting hookah smoking was to go along with their children at home. In these situations, adult women are affected by the re-emergence of traditional values and modernity. One of them said:

*“When I saw my sons go out with their friends for hookah, I told them not to go out for hookah smoking. I asked them to smoke hookah at home (Then we bought a hookah so they could smoke at home). The first time I smoked hookah in my house, it was just because I wanted my kids to smoke less and not to go out for smoking.”* (Current smoker, 47 years old, first experience at age 32)

One of the young participants said:

*“We (my husband and I) used to go out with our friends, and we smoked hookah there or smoked here (at my husband’s mother’s home). When my mom noticed my partner left our home early to smoke hookah elsewhere, she bought a hookah in order for my partner to feel comfortable in our home. Then, my dad began smoking and got addicted to hookah. My mom, too, smokes hookah occasionally. My poor mom!”* (Current smoker, 24 years old, first experience at age 9)

#### 3.1.2 The Role of Husband and His Family

About a girl or women, in addition to her parents, her sisters and brothers, her husband and her husband’s family can also influence her hookah smoking initiation. A 25-year-old participant tried hookah for the first time when she was a teenager. After marrying a man whose family smoked hookah traditionally, with the encouragement of her husband and living with her brother-in-law who was a daily smoker of hookah, she again started smoking hookah and gradually became a daily smoker:

*“We used to go out with my husband’s friends to smoke hookah while we were engaged. We rarely smoked hookah at home. After marriage, and when my brother-in-law came to live with us, he brought a hookah. Initially, we smoked every two or three nights. Little by little, it grew, and now it’s every night. On holidays, we smoke two or three times a day.”* (Current smoker, 25 years old, first experience at age 14)

One participant said about her husband’s influence on her hookah smoking initiation:

*“One night, my husband brought home a hookah. I didn’t like hookah but he said I had to prepare it for him. After one to two months (I accepted his request), and I started smoking hookah, along with my husband. We had a lot of fights. We broke a lot of hookahs (because I did not agree with my husband’s smoking of them). But it seems that we have to do everything that men want.”* (Current smoker, 32 years old, first experience at age 26)

A young woman who had started hookah smoking with other young men and women who were friends of her husband said:

*“I think my first time was when we went on a trip for fun. My husband and his friends began to smoke hookah. I said let me see what it tastes like. I just took it because I wanted to understand why my husband enjoys it. I just wanted to try it out. My husband gave it to me. Frankly speaking, I always wanted to smoke, but I felt embarrassed to do so before my husband’s friends. But that day my husband broke that feeling.”* (Current smoker, 28 years old, first experience at age 25)

A woman who began hookah smoking with her husband’s family, said:

*“The first time I smoked hookah was while we were engaged. I went to the park with my in-laws. All of them were hookah smokers. In their opinion, I was a good girl. I was sitting in a corner and didn’t smoke hookah. They told me to join them. They said, come on! Do not be too shy. My mother in-law asked me to join them, with great insistence. They urged me. Then I began. Until then I had never smoked.”* (Former smoker, 33 years old, first experience at age 25)

## 4. Discussion

This study focused on the role of family members’ smoking behaviours as a possible risk factor for initiation of hookah smoking in women. Understanding the factors that lead to the initiation of hookah smoking in women is necessary in order to formulate appropriate prevention, cessation, and policy interventions. This research is particularly important for Iranian women because among Iranian women, the use of hookah is the most common method of tobacco smoking and because of the expanded role of family in Iranian culture.

Based on the women who participated in our study, and as in the case of starting cigarette smoking (Tjora, Hetland, Aaro, & Overland, 2011; Kegler, Cleaver, & Yazzie-alencia, 2000), it has been observed that parents, siblings and peers who are hookah users can stimulate the desire of their children to smoke hookah and children often experience their first smoking in family gatherings in the presence of their parents. Since hookah is usually prepared at home or in family gatherings, girls’ participation in the preparation of hookah is inevitable and in this case they would take a few puffs. Also, the frequent observation of people, especially women who smoke hookah, plays a great role in encouraging young girls to try it. In a study by Gilman et al. (2009) about the initiation of cigarette smoking among adolescents showed that parental smoking was associated with smoking initiation in adolescent offspring. In addition, the likelihood of offspring smoking initiation increased with the number of smoking parents and the duration of exposure to parental smoking. It means frequent observation of tobacco smoking by family members is very effective in encouraging children to try hookah or cigarettes. In a few studies that have addressed this issue it has also been shown that living with a family member who used tobacco is a significant risk indicator for hookah smoking initiation (Jamil, Elsouhag, Hiller, Arnetz, & Arnetz, 2010) and family acceptance plays a role in choosing hookah over cigarettes (Weglicki, Templin, Rice, Jamil, & Hammad, 2007). In another study in Lebanon, at least 28% of first hookah trial took place with a member of the immediate family, even though the effect of friends was stronger and 58% of first hookah trial took place with a friend (Zoughaib, Adibm, & Jabbour, 2004). In the Weglicki et al study (2008), Arab-American youth reported lower percentages of cigarette smoking but significantly higher percentages of hookah smoking compared with non–Arab-American youth. Hookah smoking by family members of Arab-American youth was four times higher than family members of non–Arab-American youth. These findings are congruent with the findings of other studies reporting the important risk factor of having a family member smoking hookah at home for current hookah smoking (Barikani, 2009; Amin et al., 2012; Sarafzadegan et al., 2010).

According to our study, in addition to hookah use, the use of other tobacco products by parents, like cigarettes, can also be effective in hookah smoking initiation among girls, as the majority of participants said their father, mother or other relatives used to smoke cigarettes, and sometimes people’s tendency toward hookah smoking is rooted in their interest in cigarette smoking. According to several participants, when they were a child they had taken a few puffs of their father, mother or brother’s cigarette, hidden or in front of them, but they did not continue to use it because their parents disagreed with their cigarette smoking. In fact, based on our understanding of what the participants said, parents who are cigarette smokers are less opposed to their children smoking hookah rather than cigarettes because hookah smoking is more socially acceptable. Another reason is that when parents have poor habits regarding health, such as cigarette smoking, they cannot criticize their children habits because children copy their parent’s behavior. So, when parents smoke cigarettes it is natural that their children smoke hookah. In a study by Jamil et al. (2011) on 245 white American adults, having one or more tobacco users at home was a significant predictor of hookah use. However, the majority of hookah users (65%) had no other family members who used hookah at home, apart from themselves.

A recent systematic review has shown that in the Middle Eastern societies, expression of cultural identity is a specific motive for hookah smoking. It was particularly more acceptable for women’s use compared to cigarette smoking (AkI et al., 2013). Iranian women, too, have restrictions on cigarette smoking, although hookah use has been a traditional entertainment for many families and girls can easily make use of it at home and outside the home (Kelishadi et al., 2007). Also, our study showed that young women can smoke hookah easily in family gatherings without fear of parental reaction. But Iranian families do not accept their children smoking hookah outside or with people other than family members. Thus, they try to buy a hookah for home and encourage their children to smoke hookah only at home, not elsewhere. They believe that hookah smoking outdoors is culturally bad for girls. However, about boys, parents are more worried that when their sons are smoking hookah in hookah cafes, they may also learn to use other drugs. In fact, what parents are really worried about is that their children may smoke hookah with their friends when they are outside, not just when going to hookah cafes, because their friends may gradually teach them to smoke cigarettes or other drugs. This is the main reason why parents don’t want their children to go outside to smoke hookah. This concern may be partly true, as some reports suggest that hookah smoking is associated with substance use (Brockman et al., 2012) such as marijuana or hashish (Knishkowy & Amitai, 2005) which may be added to hookah tobacco (Shishani, Roll, & Armstrong, 2012). Also, according to Cobb, Ward, Maziak, Shihadeh and Eissenberg (2010), hookah tobacco smoking may become a gateway to the initiation of cigarette smoking. Although we should mentioned that, this is the concern of some of the parents whom we interviewed and we cannot generalize it to all parents. This finding is acquired based on conversations with several mothers and other studies should be conducted to examine it further. Indeed, whether the possibility of drug use in hookah cafes is really true or not, it must be examined in further quantitative or qualitative studies.

Another new aspect of our findings is that when parents invite children to smoke hookah at home, in order to protect them against public censure or substance abuse, an unforeseen problem may occur such that parents including the mother or other women in the family may intend to start smoking hookah. On the other hand, it may lead to the spread of hookah smoking among older female relatives. It is obvious that when something is acceptable for adult women in the families, it can easily be expanded in the community and gradually becomes part of the normal social behaviour of women. However, more research is needed to confirm this finding.

Researchers have suggested that if one person in a relationship smokes cigarettes, it is not uncommon for the partner to also be a smoker (Sutton, 1993), and especially during the transition into marriage, spouses can influence their partners’ behaviours, including their cigarette smoking behaviour (Homish & Leonard, 2005). This is also true about hookah smoking as the other new finding of our study showed that the husband of a woman has an important role in the initiation of smoking hookah, especially while they were engaged or during the first years of marriage. In fact, a man who smokes hookah may encourage his wife to smoke or his wife may be encouraged to smoke hookah in a family gathering by one of her husband’s family members including her mother-in-law. In these situations, women often accept their husband’s or his relatives’ offer because they do not want to leave their husband alone or because they do not like to be away from others. Still, some women are ashamed of hookah smoking in the presence of others, but apparently husbands try to help women to overcome this feeling of embarrassment because according to participants it has an indirect benefit for men and it is when a woman starts to smoke hookah beside her husband she would no longer criticize him for hookah smoking.

Our study has several limitations. Because of the cultural sensitivity of research issues, some participants may express only desirable social experiences and avoid expressing their real experiences. Study sampling is also a constraint which may not have the necessary distribution and the study participants may not be representative of the actual population of Iranian women. Moreover, the possibility of bias in gathering and analysis of data was another limitation. Since the researcher was not familiar with any of the study participants before the interviews and also extensive research on factors affecting the hookah smoking was not conducted, the researcher can claim that her feelings and experiences are unaffected by the data collection and analysis or this impact might have been minimal. Also, during data collection, the interviewer tried not to mention any words or give any nonverbal cues to avoid interfering, and during the analysis an attempt was made for the views of all members of the research team to be used for constructing the coding scheme.

## 5. Conclusion

We can conclude that Iranian families can have an impact on women and girls to start smoking hookah through three ways: Family traditions can lead to intention to smoke hookah in women and girls (Intergenerational transmission of family traditions, including hookah smoking); children’s smoking can lead to intention to smoke hookah in mothers (impact of the modernity of the new generation), or the husband’s and his family’s smoking behaviour can lead to intention to smoke hookah in women (impact of the modernity of the new generation and family traditions). On the other hand, intergenerational transmission of the family traditions and also unquestioned adoption of new cultural values is one of the most important factors in the initiation of hookah smoking in women and girls.

Given that the family plays an important role in the initiation of hookah use in women, tobacco use prevention and cessation interventions should be focused on targeting the family. Since some girls may try their first hookah during childhood, tobacco use prevention programs should be included in the primary school health education programs. Some behaviors that may seem to be valuable because of the cultural roots may be harmful and damaging. Thus, children should be taught that before the selection of any behaviour, they should consider its consequences. But, hookah smoking cannot be prevented only by increasing the knowledge about its dangers or consequences. Thus, one of the best ways to prevent the spread of hookah smoking is the inability to access the hookah at home. In particular, mothers can be effective in preventing the entry of hookah into homes because Iranian mothers have a large influence on the selection or exclusion of a healthy or social behaviour. Iranian families are using hookah smoking as a traditional entertainment. Thus, replacing hookah with another family pastime can reduce the spread of hookah smoking in Iranian families.

In summary, the results of this study indicate that the entry of hookah into homes can be effective in the spread of hookah smoking among adult and young women. Thus, these results may also be applied in other contexts in which family still plays a major role in the transmission of cultural values or health-related beliefs to the next generation.
